# Microgravity-Induced Alterations of Inflammation-Related Mechanotransduction in Endothelial Cells on Board SJ-10 Satellite

**DOI:** 10.3389/fphys.2018.01025

**Published:** 2018-07-31

**Authors:** Ning Li, Chengzhi Wang, Shujin Sun, Chen Zhang, Dongyuan Lü, Qin Chen, Mian Long

**Affiliations:** ^1^Key Laboratory of Microgravity – National Microgravity Laboratory, Center of Biomechanics and Bioengineering, Beijing Key Laboratory of Engineered Construction and Mechanobiology, Institute of Mechanics, Chinese Academy of Sciences, Beijing, China; ^2^School of Engineering Sciences, University of Chinese Academy of Sciences, Beijing, China

**Keywords:** microgravity, SJ-10 satellite, endothelial cells, adhesive molecules, cytoskeleton, mechanotransduction, nitric oxide, exosome RNA

## Abstract

Endothelial cells (ECs) are mechanosensitive cells undergoing morphological and functional changes in space. Ground-based study has provided a body of evidences about how ECs can respond to the effect of simulated microgravity, however, these results need to be confirmed by spaceflight experiments in real microgravity. In this work, we cultured EA.hy926 ECs on board the SJ-10 Recoverable Scientific Satellite for 3 and 10 days, and analyzed the effects of space microgravity on the ECs. Space microgravity suppressed the glucose metabolism, modulated the expression of cellular adhesive molecules such as ICAM-1, VCAM-1, and CD44, and depressed the pro-angiogenesis and pro-inflammation cytokine secretion. Meanwhile, it also induced the depolymerization of actin filaments and microtubules, promoted the vimentin accumulation, restrained the collagen I and fibronectin deposition, regulated the mechanotransduction through focal adhesion kinase and Rho GTPases, and enhanced the exosome-mediated mRNA transfer. Unlike the effect of simulated microgravity, neither three-dimensional growth nor enhanced nitric oxide production was observed in our experimental settings. This work furthers the understandings in the effects and mechanisms of space microgravity on ECs, and provides useful information for future spaceflight experimental design.

## Introduction

Endothelial cells (ECs) form the inner lining of blood vessels and play a key role in maintaining blood-tissue barrier and controlling vascular permeability and immune cell trafficking (Danese et al., 2007; Dorland and Huveneers, 2017). ECs are highly sensitive to mechanical forces such as fluid shear stress, cyclic tensile strain and substrate stiffness, and contribute to cardiovascular deconditioning and immune dysfunction faced by astronauts upon the removal of gravitational forces during spaceflight (Byfield et al., 2009; Chancellor et al., 2010; Maier et al., 2015). Exposure to microgravity in space can induce the morphological and gene expression changes in ECs, displaying the heterogeneity of cell size and shape (Kapitonova et al., 2012), three-dimensional growth (Pietsch et al., 2017), energy and protein metabolism deficiency (Chakraborty et al., 2018), significant suppression of genes associated with host defense (Chakraborty et al., 2014), and alterations in genes involved in cell adhesion, oxidative phosphorylation, and stress responses (Versari et al., 2013). To date, only a few relevant studies in space have been reported and the impact of real microgravity on EC functions is still unclear mainly due to the rareness and high costs of spaceflight missions.

Ground-based microgravity effect simulation facilities, including rotating wall vessel (RWV) (a kind of two-dimensional (2D) clinostat) (Goodwin et al., 1993), random positioning machine (RPM) (a kind of three-dimensional (3D) clinostat) (Wuest et al., 2017) and other types of clinostats (Siamwala et al., 2010; Kang et al., 2011), have been devoted to unravel the effect of time-averaged gravity vector on EC functions in more details. Specifically in the cascade of leukocyte recruitment onto endothelium, not only the adhesive receptor expression and chemokine secretion of neutrophils are altered in RWV (Wang et al., 2015) but the adhesive ligand expression and inflammatory cytokine secretion of ECs are also modulated by this kind of simulation. Unfortunately, the outcomes are still controversial in the literatures. For instance, the expression of intercellular adhesion molecule-1 (ICAM-1) on non-activated ECs is decreased significantly when cultured in RPM for 24 h (Grenon et al., 2013). However, ICAM-1 mRNA is up-regulated after 30 min clinorotation and the clustering of ICAM-1s on cell membrane is observed when ECs are activated by TNF-α and cultured in RWV (Zhang et al., 2010). Mechanistically, cellular responses to microgravity are a typical mechano-biological process and highly associated with cytoskeletal remodeling (van Loon, 2009; Long et al., 2015). The reorganization of cytoskeletons including microtubules and actin filaments (F-actin) are also known to be regulated by the effect of simulated microgravity (Buravkova et al., 2018) as well as by gravity vector orientation (Zhang et al., 2017; Zhou L.W. et al., 2018). Markedly down-regulated amount of actin (Carlsson et al., 2003), depolymerization of F-actin (Kang et al., 2011), and clustering of the stress fibers at the cellular membrane and around the nucleus (Infanger et al., 2007; Grenon et al., 2013) are presented, and typically the actin rearrangement is RhoA-dependent (Shi et al., 2017). Cytoskeletal remodeling was accompanied by the overexpression of extracellular matrix (ECM) proteins, including collagen I, fibronectin, osteopontin, and laminin (Infanger et al., 2006a; Grimm et al., 2009; Buravkova et al., 2018). Furthermore, intracellular signaling and cell–cell communication are also crucial to EC functions in microgravity. For example, nitric oxide (NO), an important second messenger and vasodilator produced by endothelial nitric oxide synthase (eNOS), is shown to be up-regulated by RPM and is deemed to be responsible for angiogenesis and cardiovascular deconditioning experienced by astronauts during spaceflight (Siamwala et al., 2010; Grenon et al., 2013). Phosphoinositide 3-kinase (PI3K) pathway (Shi et al., 2012), actin remodeling (Siamwala et al., 2010) and caveolin-1 (Cav-1)-mediated mechanotransduction (Shi et al., 2016) can act as the upstream signaling of eNOS activation by 2D or 3D clinostat culture in ECs. Thus, these results present clues to understand how ECs function in simulated microgravity effects.

While these microgravity effect simulations provide an insight into optimizing future spaceflight missions, they cannot replace spaceflight experiments yet. On one hand, spaceflight is more complicated where microgravity is combined with hypergravity, radiation, and vibration (Kapitonova et al., 2012). On the other hand, the kinematic rotation of these simulating devices can induce fluid motion in the cell chamber and mediate extra forces to the cells cultured (Wuest et al., 2017). Therefore, the results obtained using these techniques should be confirmed by space experiments in real microgravity.

Here, we systematically examined the effects of real space microgravity on ECs and analyzed the underlying mechanisms on board the SJ-10 Recoverable Scientific Satellite. Cellular morphology, metabolism, adhesive molecule expression, cytokine secretion, cytoskeletal remodeling, ECM accumulation, NO production, and the related mechanotransduction pathways were compared between EA.hy926 cells cultured in space and on ground. The exosome-mediated mRNA transfer and cell–cell communication were also discussed.

## Materials and Methods

### Antibodies

Alexa Fluor^®^647-conjugated mouse anti-ICAM-1 (HCD54) monoclonal antibodies (mAbs) were obtained from BioLegend (San Diego, CA, United States). Alexa Fluor^®^594-conjugated rabbit anti-β-actin (13E5), Alexa Fluor^®^555-conjugated rabbit anti-α-tubulin (11H10) and Alexa Fluor^®^647-conjugated rabbit anti-vimentin (D21H3) mAbs were purchased from Cell Signaling Technology (Danvers, MA, United States). Alexa Fluor^®^647-conjugated rabbit anti-VCAM-1 (EPR5047) and anti-Cav-1 (E249) mAbs, Alexa Fluor^®^488-conjugated rabbit anti-fibronectin (F1) and mouse anti-β1-integrin (12G10) mAbs, PE-conjugated mouse anti-CD44 (F10-44-2) mAbs, rabbit anti-vinculin (EPR8185) and anti-Rho-associated coiled-coil kinase-1 (ROCK-1, EP786Y) mAbs, mouse anti-Rac-1 (0.T.127), anti-phospho-focal adhesion kinase (p-FAK, Tyr^397^, M121), anti-RhoA (1B12), and PI3K-p85 (M253) mAbs, rabbit anti-collagen I, anti-eNOS, and anti-Cdc42 polyclonal antibodies, DyLight^®^594-conjugated donkey anti-rabbit and Alexa Fluor^®^488-conjugated goat anti-rabbit and donkey anti-mouse polyclonal secondary antibodies were all from Abcam (Cambridge, United Kingdom).

### Cell Culture and Spaceflight Mission Procedure

EA.hy926 ECs constructed by fusing primary human umbilical vein cells with lung adenocarcinoma cell line A549 clone were purchased from American Type Culture Collection (ATCC, Manassas, VA, United States) and cultured in Endothelial Cell Medium (Sciencell Research Laboratories, Carlsbad, CA, United States) at 37°C with 5% CO_2_.

**FIGURE 1:**
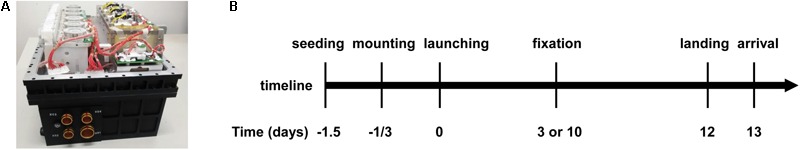
Overview of the hardware and timeline of the task at SJ-10 Recoverable Scientific Satellite. **(A)** Photo of the space cell culture system (SCCS) hardware. **(B)** Timeline of the entire mission procedure of SJ-10 satellite inside the SCCS, indicating cell seeding, culture chamber mounting, satellite launching, cell fixation, return of the re-entry module to Earth, and arrival of the cell chambers at the laboratory.

The spaceflight experiment was performed in a space cell culture system (SCCS, **Figure 1A**) developed in-house as a payload of the SJ-10 satellite mission launched on 6th April and recovered on 18th April, 2016 (**Figure 1B**) (Hu et al., 2014). Thirty-six hours before the launch, two cell culture chambers with an effective culture area of 12 cm^2^ each were filled with a cell suspension containing 5 × 10^5^ EA.hy926 cells per chamber and placed in an incubator for 12 h to allow cell attachment at 37°C. Nineteen hours before launching, the two chambers were re-filled with fresh medium to wash out the un-attached cells and to eliminate air bubbles, and assembled in the SCCS. The SCCS was then inflated by pre-mixed 95% air and 5% CO_2_ sealed at 1 atm and mounted onto the platform of the SJ-10 satellite at 8 h pre-launch. Gas exchange between culture medium and environment inside the SCCS was available through a silicon rubber seal in the culture chamber and the connected silicon rubber tubes. The cells in the two chambers were maintained at a temperature of 36 ± 1.5°C and fixed with 4% paraformaldehyde on orbit at day 3 or day 10 post-launch, respectively. The medium for the sample fixed at day 10 was refreshed every 48 h before fixation but not refreshed for the sample fixed at day 3, and the supernatants in the two chambers were collected separately and preserved at 5–15°C. A total of 3 or 12 mL supernatant was obtained for the ECs cultured for 3 or 10 days, respectively. All the in-orbit operations were performed automatically by a set of peristaltic pumps and pinch valves controlled by preset programming codes. After running in orbit for 12 days, the re-entry module of the satellite was initiated for returning it to the ground. The cell chambers and medium samples were taken out from the SCCS and transported at 4–10°C to the laboratory in Beijing within 18 h. The cell culture substrates (Permanox^®^slide, 25 cm × 75 cm, Nunc, Roskilde, Denmark) were taken apart from the chambers and washed three times with Dulbecco’s phosphate-buffered saline (DPBS) and the supernatants were stored at -70°C immediately until test. The corresponding ground control experiments were conducted four times independently using the same SCCS hardware and following the same timeline procedure as the spaceflight experiment did. The cell culture temperature, sample preservation condition, timing of cell seeding, SCCS mounting, medium refreshing, cell fixation and sample recovery were in strict accordance with the in-flight experiment.

### Optical and Scanning Electron Microscope (SEM) Imaging

The EA.hy926 cells placed on the entire cell culture substrate were observed by an Olympus inverted microscope (CKX41, Waltham, MA, United States) with a 10×/NA 0.25 objective and recorded with a charge-coupled device (CCD) camera. Each chamber substrate was then cut into pieces and used for SEM imaging or immunofluorescence (IF) staining. The samples for SEM observation were dehydrated with ethanol gradient (50%, 70%, 80%, 90%, and 100%) for 5 min in each concentration, transferred to tert-butyl alcohol for two changes, dried at critical point, coated with a thin layer of gold, and imaged by FEI Quanta 200 SEM (Hillsboro, OR, United States). Other samples for IF staining were described below.

### IF Staining

Cellular adhesive molecules and ECM proteins from collected cells were stained without permeabilization. For intracellular signaling molecules and cytoskeletal protein components, the fixed cells were permeabilized with 0.4% Triton X-100 in DPBS for 15 min and incubated successively in blocking buffer (1% BSA in DPBS), primary antibodies (10 μg/mL in blocking buffer) and secondary antibodies (5 μg/mL in blocking buffer) for 1 h at 37°C. After each step, the cells were washed three times in DPBS. The images of stained cells were collected using a Zeiss LSM710 confocal microscope system (Zeiss, Oberkochen, Germany) with a 63× oil immersion objective.

### Cell Metabolism Analysis, Cytokine Arrays, and NO Production Test

Glucose and L-lactate concentrations in the collected supernatants were determined by commercial glucose (Huaxingbio, Beijing, China) and L-lactate (BioAssay Systems, Hayward, CA, United States) assay kits following the manufacturers’ protocols, respectively.

Cytokine arrays were performed using the Proteome Profiler Human XL Cytokine Array Kit (R&D Systems, Minneapolis, MN, United States) to detect 102 different cytokines in the supernatants, according to the manufacturer’s protocol. For each assay, a total of 500 μL of cell culture supernatant was used. Quantitative analysis of spot density was obtained using ImageJ software (National Institutes of Health, Bethesda, MD, United States).

The levels of total NO, nitrate (NO_3_^-^) and nitrite (NO_2_^-^) were analyzed in the supernatants by Griess reaction using a colorimetric assay kit from R&D systems upon the manufacturer’s protocol. NO level was determined by measuring absorbance at 540 nm using a microplate reader (iMark, Bio-Rad, Hercules, CA, United States).

### Exosome RNA Profiling

A total of 10 mL supernatant was used for exosome collection through standard centrifugation steps as previously described (Théry et al., 2006). Total RNA from purified exosomes was used for library preparation and sequencing performed at RiboBio (Guangzhou, China). Briefly, total RNA was reversely transcribed and amplified by PCR. The PCR products were sequenced using the Illumina HiSeq 3000/4000 platform (San Diego, CA, United States). The sequence data was upload to the Sequence Read Archive (SRA)^1^ and mapped to human reference genome with Tophat2 (Kim et al., 2013). Gene expression was quantified with RPKM values (Reads Per Kilobase of transcript sequence per Millions base pairs sequenced) (Mortazavi et al., 2008) and the differential expression was assessed by DEseq (Anders and Huber, 2010). Differentially expressed genes were chosen according to the criteria of fold change > 2 and *P*-value < 0.05. Gene ontology (GO) analysis was applied to analyze the potential functions of the differentially expressed genes using DAVID (Database for Annotation, Visualization and Integrated Discovery) (Ashburner et al., 2000). The significance of GO term was indicated by *P*-value and *P* < 0.05 was considered to be statistically significant.

### Data Availability Statement

The raw data from this study will be made available by the authors, without undue reservation, to any qualified researcher.

### Statistical Analysis

Data were presented as means ± SEM. The significant differences between multiple groups were analyzed by two-way analysis of variance (ANOVA), followed by Holm–Sidak test. For two group comparisons, the unpaired two-tailed Student’s *t-*test was performed if passing the normality test or using Mann–Whitney rank sum test if not. A *P*-value less than 0.05 was considered statistically significant.

## Results

### Promoting Morphological Alteration in Space

**FIGURE 2:**
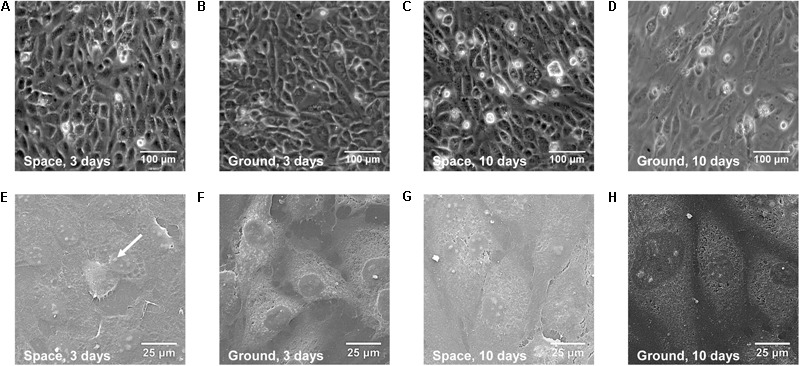
Morphology of EA.hy926 cells cultured in space or on ground. Presented were the optical **(A–D)** and SEM **(E–H)** images of EA.hy926 cells cultured for 3 **(A,B,E,F)** or 10 **(C,D,G,H)** days in space **(A,C,E,G)** and on ground **(B,D,F,H)**. *Arrow* indicated a bleb.

To evaluate whether real space microgravity affects EC morphology and function, we cultured EA.hy926 cells in space and on ground for 3 and 10 days (**Figure 2**). The cells cultured in space (**Figures 2A,C**) displayed similar spindle-like shape and formed a confluent monolayer as the control cells on ground (**Figures 2B,D**). We neither observed microgravity-induced groove or tube-like structures nor multi-cellular spheroids formed by EA.hy926 cells as those previous studies described in space or on ground (Grimm et al., 2009; Ma et al., 2014; Aleshcheva et al., 2016; Pietsch et al., 2017). SEM imaging provided more details on the structure of the cells (**Figures 2E–H**) and supported the above optical observations. Evidently, space microgravity led to the flatter cells with less obvious nuclei and more blebs on cell membrane.

### Suppressing Energy Metabolism

**FIGURE 3:**
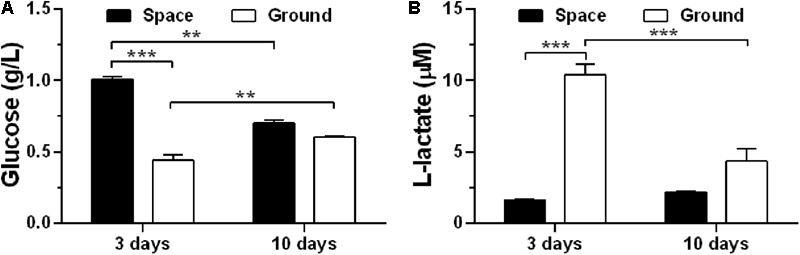
Effects of space microgravity on cell metabolism. Glucose **(A)** and L-lactate **(B)** concentrations were determined from the supernatant of EA.hy926 cells cultured for 3 or 10 days in space (*closed bars*) and on ground (*open bars*). Data were presented as the mean ± SEM of one experiment in space and two to four independent experiments on ground, both of which were performed in technical triplicate and analyzed with two-way ANOVA followed by Holm–Sidak test. ^∗^*P* < 0.05; ^∗∗^*P* < 0.01; ^∗∗∗^*P* < 0.001.

To assess if exposure of ECs to space microgravity has a significant impact on cell energy metabolism, we quantified the glucose (**Figure 3A**) and L-lactate (**Figure 3B**) concentrations from the cell culture supernatants. Compared with ground control, the glucose concentration increased additional 129% (**Figure 3A**) and the L-lactate concentration was reduced to 16% (**Figure 3B**) for EA.hy926 cells cultured for 3 days in space, implying a suppressed metabolism with lower glucose consumption and lactate production in agreement with previous study (Chakraborty et al., 2018). The significant difference disappeared between the cells cultured for 10 days in space and on ground (**Figure 3**), probably due to the slowed growth associated with contact inhibition in long-term space culture.

### Mediating EC Immunodeficiency

**FIGURE 4:**
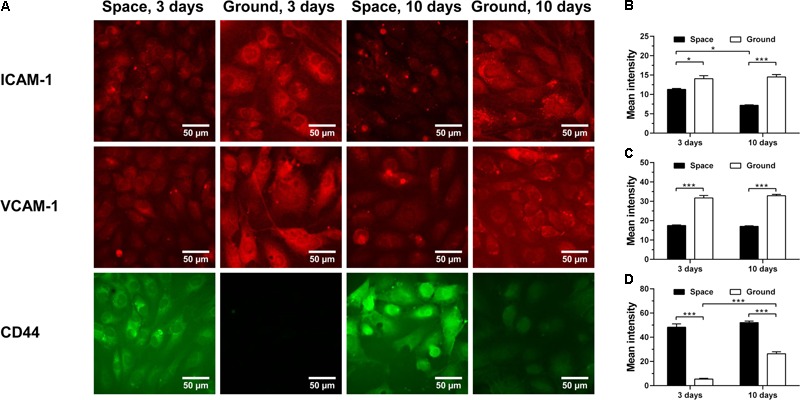
Effects of space microgravity on expression of adhesive molecules. Plotted are typical immunofluorescent images **(A)** and measured mean intensity **(B–D)** of ICAM-1 **(B)**, VCAM-1 **(C)**, and CD44 **(D)** on EA.hy926 cells cultured for 3 or 10 days in space (*closed bars*) and on ground (*open bars*). Data were presented as the mean ± SEM of 20–80 images from one experiment in space and three to four independent experiments on ground and analyzed with two-way ANOVA followed by Holm–Sidak test. ^∗^*P* < 0.05; ^∗∗^*P* < 0.01; ^∗∗∗^*P* < 0.001.

**FIGURE 5:**
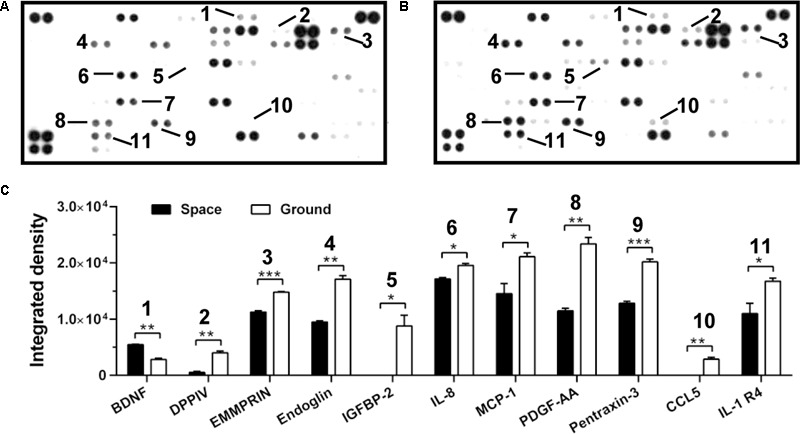
Effects of space microgravity on cytokine secretion. Plotted are cytokine array analyses of the supernatant of EA.hy926 cells cultured for 10 days in space **(A)** and on ground **(B)**. The lines with numbers marked those cytokines that were significantly regulated. **(C)** Integrated pixel densities of each marked spot detected in the cytokine array were quantified by ImageJ and represented as a bar graph for cells cultured in space (*closed bars*) and on ground (*open bars*). Data were presented as the mean ± SEM of one experiment in space and two independent experiments on ground both of which were performed in technical duplicate and analyzed with unpaired two-tail Student’s *t-*test or Mann–Whitney rank sum test. ^∗^*P* < 0.05; ^∗∗^*P* < 0.01; ^∗∗∗^*P* < 0.001.

The energy deficiency caused by spaceflight is potentially cascaded into dysregulation of protein metabolism and impairment of host immunity (Chakraborty et al., 2018). Here, we analyzed the effects of space microgravity on expression of adhesive molecules (**Figure 4**) and cytokine secretion (**Figure 5**). ICAM-1 and vascular cell adhesion molecule-1 (VCAM-1) are the ligands for β2 and α4 integrins, respectively, that are responsible for leukocyte recruitment and transmigration during inflammation. Space microgravity could significantly decrease the presence of ICAM-1 (**Figure 4A** *1st row* and **Figure 4B**) and VCAM-1 (**Figure 4A** *2nd row* and **Figure 4C**) on EC surface at 3 and 10 days. However, the expression of CD44, another important adhesive molecule that interacts with hyaluronan (HA) in ECM to regulate EC proliferation and angiogenesis (Savani et al., 2001), was 8.9- and 2.0-fold increased in the presence of space microgravity (**Figure 4A** *3rd row* and **Figure 4D**). Such the reverse expressions between ICAM-1/VCAM-1 and CD44 implied that the effect of space microgravity on different adhesive molecule expression is diverse and CD44 could compensate the reduced cell adhesion mediated by ICAM-1/VCAM-1.

Cytokine secretion and chemokine production are also crucial to regulate the cellular responses in space (Chakraborty et al., 2014). Here, the secretion of ten cytokines and chemokines (DPPIV, EMMPRIN, Endoglin, IGFBP-2, IL-8, MCP-1, PDGF-AA, Pentraxin-3, CCL5, and IL-1 R4) was lower in ECs exposed to space microgravity for 10 days than in ground control cells, whereas the expression of only one cytokine (BDNF) was slightly higher (**Figure 5**), indicating the suppressed function in angiogenesis and inflammation.

### Inducing Cytoskeletal Remodeling

**FIGURE 6:**
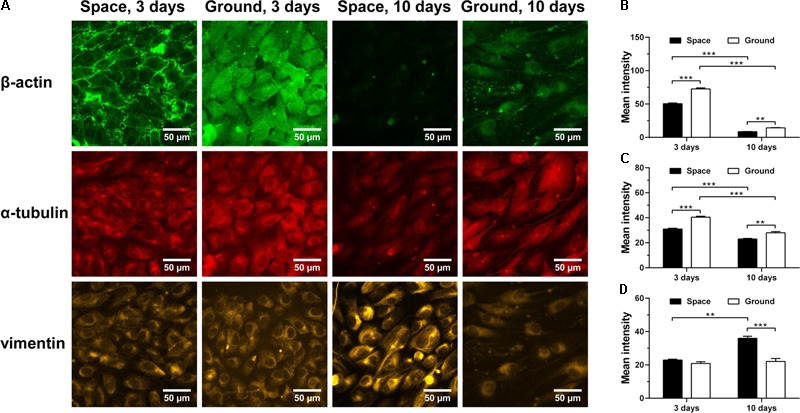
Effects of space microgravity on cytoskeletal remodeling. Plotted are typical immunofluorescent images **(A)** and measured mean intensity **(B–D)** of β-actin **(B)**, α-tubulin **(C)**, and vimentin **(D)** in EA.hy926 cells cultured for 3 or 10 days in space (*closed bars*) and on ground (*open bars*). Data were presented as the mean ± SEM of 20–80 images from one experiment in space and three to four independent experiments on ground and analyzed with two-way ANOVA followed by Holm–Sidak test. ^∗^*P* < 0.05; ^∗∗^*P* < 0.01; ^∗∗∗^*P* < 0.001.

Cytoskeletal rearrangement of ECs has been observed in both real microgravity (Kapitonova et al., 2012) and microgravity effect simulation (Carlsson et al., 2003; Infanger et al., 2007; Kang et al., 2011; Grenon et al., 2013). Here, we found that 3-day culture in space led to a significant decrease in the amount of F-actin (**Figure 6A** *1st row* and **Figure 6B**) and microtubules (**Figure 6A** *2nd row* and **Figure 6C**). This response remained similar after 10 days of exposure to space microgravity, although the absolute expressions of actin and tubulin were reduced drastically compared with those in ECs cultured for 3 days. This observation is possibly attributed to long-term contact inhibition with slowed growth, which can negatively alter the cytoskeletal elements necessary for cell morphology and motility (Everding et al., 2000; Hung and Terman, 2011). In addition to actin depolymerization, space microgravity also induced F-actin accumulation at the cellular membrane at 3 days (**Figure 6A** *1st row*). Intriguingly, the expression of vimentin remained similar to the ground control after exposure to space microgravity for 3 days and increased 63% after 10 days (**Figure 6A** *3rd row* and **Figure 6D**), which might compensate the loss of mechanical stability caused by F-actin and microtubule remodeling especially in long-term culture (Zhang et al., 2017; Zhou L.W. et al., 2018).

### Restraining ECM/Integrin/FAK Pathway

**FIGURE 7:**
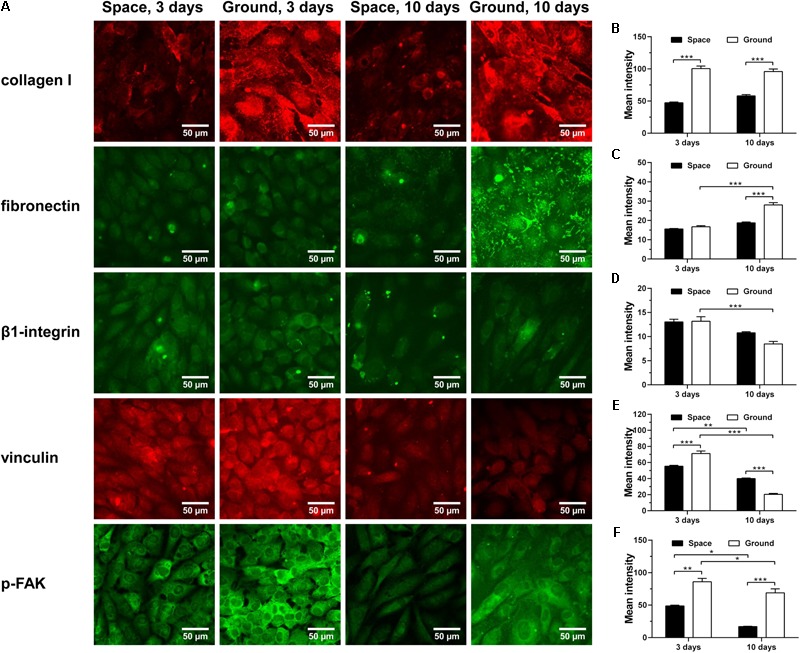
Effects of space microgravity on ECM/integrin/FAK pathway. Plotted are typical immunofluorescent images **(A)** and measured mean intensity **(B–F)** of collagen I **(B)**, fibronectin **(C)**, β1-integrin **(D)**, vinculin **(E)**, and p-FAK **(F)** expressed by EA.hy926 cells cultured for 3 or 10 days in space (*closed bars*) and on ground (*open bars*). Data were presented as the mean ± SEM of 20–80 images from one experiment in space and three to four independent experiments on ground and analyzed with two-way ANOVA followed by Holm–Sidak test. ^∗^*P* < 0.05; ^∗∗^*P* < 0.01; ^∗∗∗^*P* < 0.001.

ECM/integrin/FAK pathway plays a crucial role in mechanotransduction, providing a functional linkage between ECM stiffness and cytoskeletal remodeling (Provenzano and Keely, 2011). Collagen I and fibronectin are two major ECM components with Arg-Gly-Asp (RGD) sequence binding to β1-integrin receptors on cellular membrane. Here, significantly decreased expression of collagen I was observed for ECs cultured in space for 3 and 10 days (**Figure 7A** *1st row* and **Figure 7B**), while reduced production of fibronectin was only found in cells exposed to space microgravity for 10 days (**Figure 7A** *2nd row* and **Figure 7C**). The expression of β1-integrin after exposure to space microgravity remained unchanged compared to the ground control (**Figure 7A** *3rd row* and **Figure 7D**), but significant changes were observed for focal adhesion scaffolding protein vinculin with a 22% decrease at 3 days and a 96% increase at 10 days, respectively (**Figure 7A** *4th row* and **Figure 7E**). Moreover the tyrosine phosphorylation of FAK in ECs was reduced to 25%-57% of ground control (**Figure 7A** *5th row* and **Figure 7F**), indicating inhibited activation of ECM/integrin/FAK pathway by spaceflight. Taken together, this mechanotransductive pathway is suppressed in space microgravity.

### Presenting Diverse Rho GTPase Expression

**FIGURE 8:**
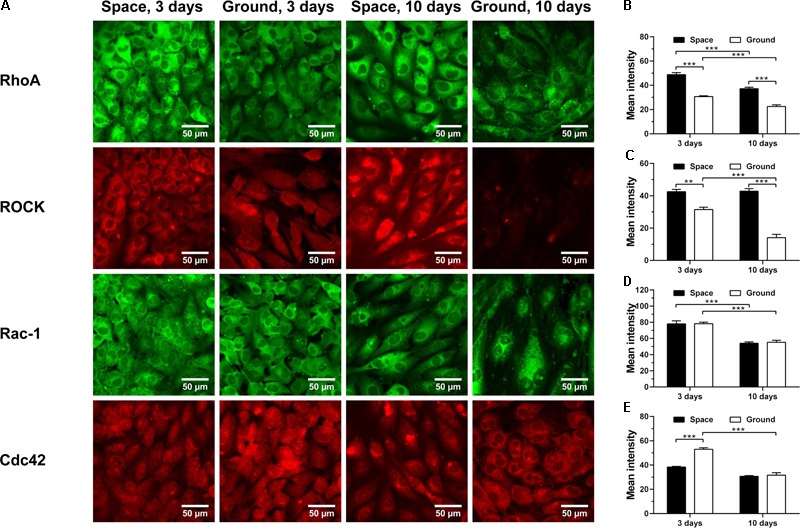
Effects of space microgravity on the expression of Rho GTPases and ROCK. Plotted are typical immunofluorescent images **(A)** and measured mean intensity **(B–E)** of RhoA **(B)**, ROCK **(C)**, Rac-1 **(D)**, and Cdc42 **(E)** in EA.hy926 cells cultured for 3 or 10 days in space (*closed bars*) and on ground (*open bars*). Data were presented as the mean ± SEM of 20–80 images from one experiment in space and three to four independent experiments on ground and analyzed with two-way ANOVA followed by Holm–Sidak test. ^∗^*P* < 0.05; ^∗∗^*P* < 0.01; ^∗∗∗^*P* < 0.001.

RhoA, Rac-1 and Cdc42 are the three members of Rho GTPases family, important for the rearrangement of cytoskeletal actin, tubulin and vimentin and regulated by integrin-stimulated phosphorylation of FAK or hyaluronan–CD44 interaction (Bourguignon et al., 2005, 2006; Cau and Hall, 2005; Grigoriev et al., 2006; Torre et al., 2010; Provenzano and Keely, 2011; Chemin et al., 2012; Schofield et al., 2012; Chen et al., 2013; Murray et al., 2014; Hyder et al., 2015; Rozés Salvador et al., 2016; Shi et al., 2017; Yang et al., 2017). Here, spaceflight-induced enhancement of RhoA (**Figure 8A** *1st row* and **Figure 8B**) and downstream signaling molecule ROCK (**Figure 8A** *2nd row* and **Figure 8C**) was found at 3 and 10 days, presumably stemming from the increased expression of CD44 (**Figure 4D**). The expression of Rac-1 was unaffected by space microgravity (**Figure 8A** *3rd row* and **Figure 8D**). The expression of Cdc42 was significantly decreased after 3 days of exposure to space microgravity (**Figure 8A** *4th row* and **Figure 8E**), consistent with the inhibition of ECM/integrin/FAK pathway (**Figure 7**). These diverse expressions of distinct Rho GTPase members implied the complicated signaling network for the ECs in space.

### Maintaining Similar NO Production

**FIGURE 9:**
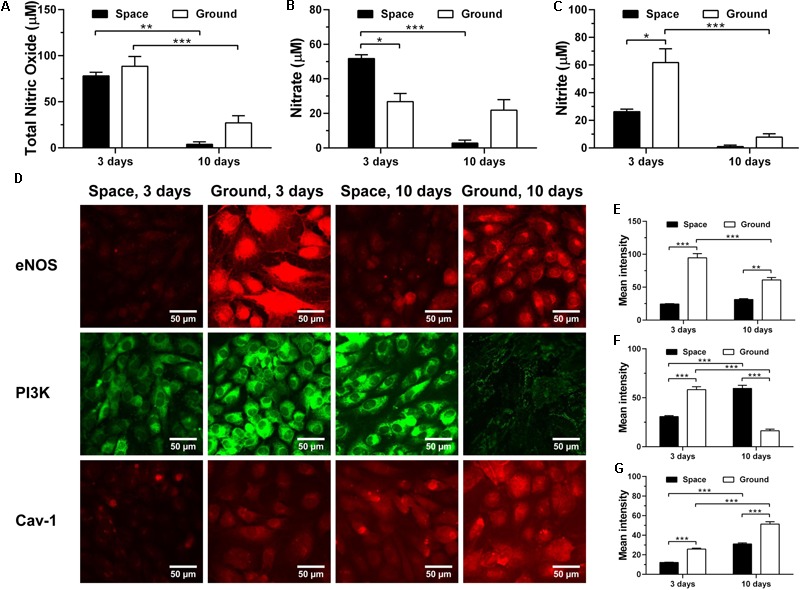
Effects of space microgravity on production of nitric oxide (NO) and expressions of Cav-1, eNOS, and PI3K. Total NO **(A)**, nitrate **(B)**, and nitrite **(C)** concentrations were determined from the supernatant of EA.hy926 cells cultured for 3 or 10 days in space (*closed bars*) and on ground (*open bars*). Plotted are typical immunofluorescent images **(D)** and measured mean intensity **(E–G)** of eNOS **(E)**, PI3K **(F)**, and Cav-1 **(G)** in EA.hy926 cells cultured for 3 or 10 days in space (*closed bars*) and on ground (*open bars*). Data were presented as the mean ± SEM of three to nine samples **(A–C)** or 20–80 images **(E–G)** from one experiment in space and two to four independent experiments on ground and analyzed with two-way ANOVA followed by Holm–Sidak test. ^∗^*P* < 0.05; ^∗∗^*P* < 0.01; ^∗∗∗^*P* < 0.001.

To test whether space microgravity affects NO production of ECs, we determined the total NO, nitrate and nitrite release (**Figures 9A–C**) and the changes of related regulating proteins (**Figures 9D–G**). The NO production remained at low levels no matter if the ECs were cultured in space or on ground (**Figures 9A–C**), especially at 10 days. The total NO was not modulated by space microgravity (**Figure 9A**), since the significant increase of nitrate (**Figure 9B**) counteracted the decrease of nitrite at 3 days (**Figure 9C**). The expression of eNOS was reduced to 26% of ground control in the presence of space microgravity for 3 days (**Figure 9D** *1st row* and **Figure 9E**), which might be induced by the decreased PI3K (**Figure 9D** *2nd row* and **Figure 9F**). After 10 days of exposure to space microgravity, significantly enhanced PI3K was found compared with ground control (**Figure 9D** *2nd row* and **Figure 9F**), and the amount of eNOS was recovered to 51% of ground control (**Figure 9D** *1st row* and **Figure 9E**). Despite the down-regulated expression of eNOS, the Cav-1 was depressed by space microgravity (**Figure 9D** *3rd row* and **Figure 9G**), which could release more unanchored eNOS and enhance eNOS activity (Shi et al., 2016). Thus, real microgravity shows no effects to enhance the production of NO as the clinostat does on ground (Siamwala et al., 2010; Grenon et al., 2013).

### Enhancing Exosome mRNA Transfer

**FIGURE 10:**
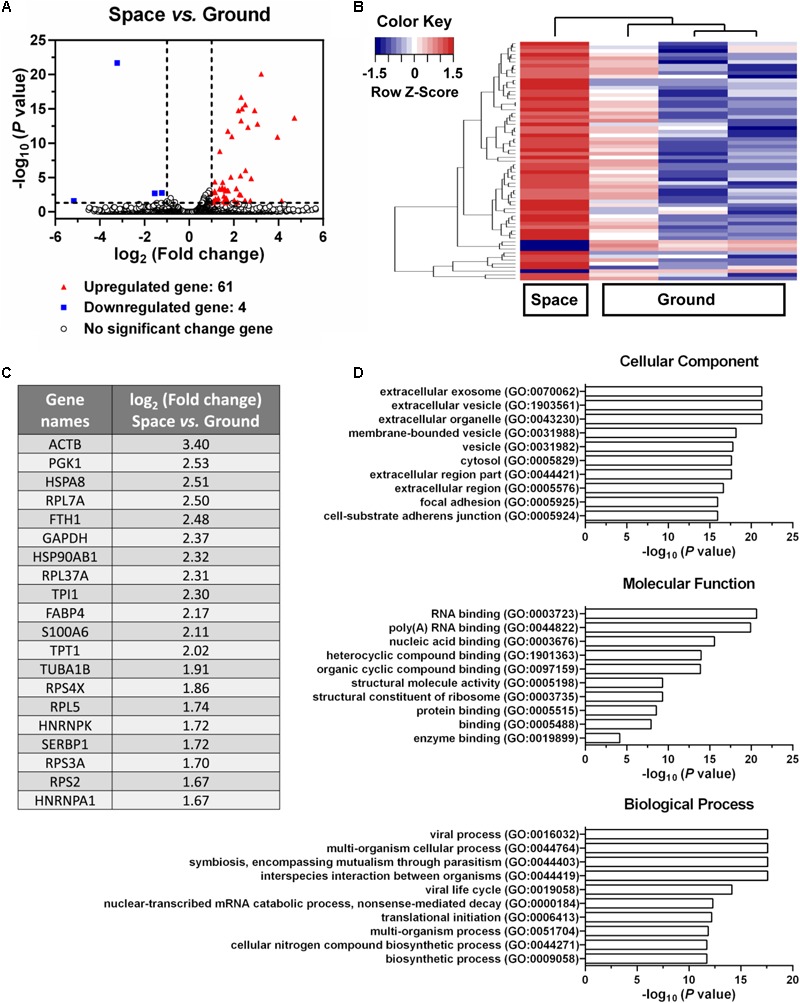
Effects of space microgravity on RNA profile in exosomes collected from the supernatant of EA.hy926 cells cultured for 10 days in space and on ground. **(A)** Volcano plots of exosome RNA profile (Space vs. Ground). The *horizontal line* represents a *P*-value of 0.05 and the two *vertical lines* correspond to twofold up or down. The *red* and *blue* points represent the up-regulated and down-regulated genes with statistical significance respectively. **(B)** Hierarchical clustering analysis of significantly changed genes (Space vs. Ground). The relative abundance of a gene expression in a given sample was colored by its row Z-score, calculated by subtracting the mean expression across all samples from its value for a given sample and then dividing by the standard deviation across all the samples. Each *row* denotes a gene; the *columns* represent one experiment in space and three independent experiments on ground. *Red*, increased expression; *blue*, decreased expression. **(C)** The top 20 up-regulated protein coding genes in exosomes from EA.hy926 cells cultured in space compared to that on ground. **(D)** Gene ontology (GO) term enrichment and pathway analysis of the differentially expressed genes. The top 10 enriched GO terms in cellular component, molecular function and biological process were calculated by the value of –log_10_ (*P*-value) and presented in bar diagrams.

Endothelial cells are capable of transferring genetic information to the neighboring cells through the release of exosomes, which further presents an important role in inflammation, atherosclerosis, and angiogenesis (Loyer et al., 2014). To evaluate whether space microgravity can affect exosome-mediated genetic material transfer and the related cell–cell communication, the exosomes were collected from the supernatants of ECs cultured in space and on ground, and the RNA profiles in the exosomes were compared (**Figure 10**). 61 genes were up-regulated in space microgravity group compared to ground control group, while only four genes were down-regulated (**Figures 10A,B**). The top 20 up-regulated protein coding genes in the exosomes from ECs cultured in space compared to that on ground were presented in **Figure 10C**, including the ACTB and TUBA1B gene encoding β-actin and tubulin α-1B, respectively. Moreover, the GO analysis was performed to determine the gene enrichment in cellular components, molecular functions and biological processes (**Figure 10D**). The differentially expressed genes were frequently enriched in such cellular components as extracellular exosome, focal adhesion, and cell-substrate adherens junction, involved in the molecular functions of RNA binding, structural molecule activity and protein binding, and took part in the biological processes like viral process, multi-organism cellular process and biosynthetic process. These results demonstrated that space microgravity could enhance the mRNA transfer and cell–cell communication among ECs.

## Discussion

**FIGURE 11:**
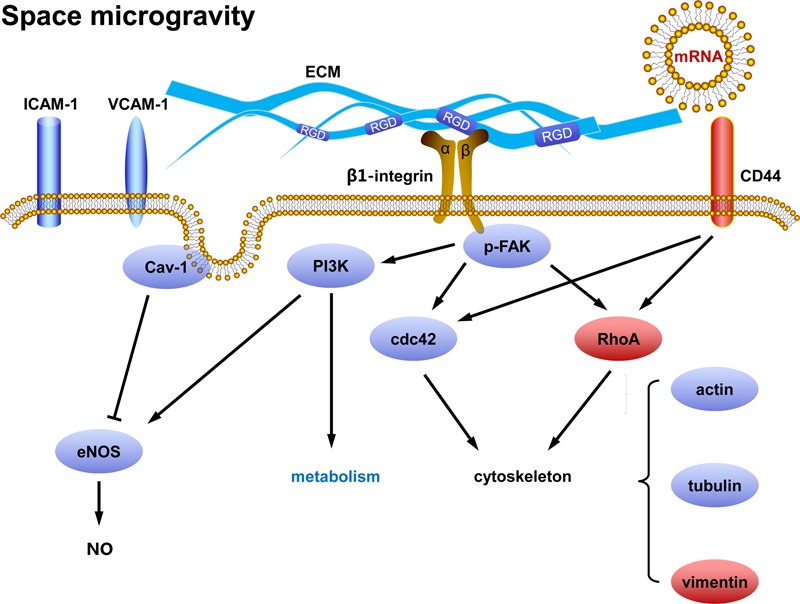
Working model for the effects of space microgravity on endothelial cells. *Red* and *blue* icons represented up- and down-regulated molecules, respectively.

This work aims to elucidate how ECs respond to real microgravity and verify the validity of the conclusions drawn from ground-based microgravity effect simulation. Our data demonstrate that space microgravity suppress energy metabolism, cytokine secretion and ECM expression, modulate distinctly adhesive molecule expression, induce cytoskeletal remodeling, and enhance exosome-mediated mRNA transfer (**Figure 11**). Our results conformed several major findings demonstrated by those studies on ECs in microgravity effect simulation using clinostats, such as decreased cell-surface expression of ICAM-1 and VCAM-1 (Grimm et al., 2010; Grenon et al., 2013), reduced release of pro-inflammatory cytokines (Cotrupi et al., 2005; Grimm et al., 2010; Griffoni et al., 2011; Grenon et al., 2013) and disorganization of F-actin and microtubules (Kang et al., 2011; Janmaleki et al., 2016). However, unlike the effect of simulated microgravity, space microgravity neither enhanced NO production nor induced 3D growth in the current work, indicating a different role in angiogenesis between the two approaches.

Angiogenesis, a process of new blood vessels forming from the existing blood vessels, is known to be promoted by the effect of simulated microgravity in previous studies. On one hand, ECs cultured in clinostats exhibit significantly increased capacities in both cell migration and tube formation (Siamwala et al., 2010), depending on the increased NO production (Shi et al., 2012) and decreased Cav-1 (Shi et al., 2016). Down-regulation of Cav-1 expression and up-regulation of PI3K-Akt signal pathway are both engaged in promoting the activity of eNOS and NO-related angiogenesis. 3D growth of ECs in clinostat culture is another marker for enhanced angiogenesis (Grimm et al., 2009; Ma et al., 2014; Aleshcheva et al., 2016; Pietsch et al., 2017). Tubular structures and multicellular spheroids are formed after long-term clinostat culture, accompanying with increased expression of ECM proteins (Infanger et al., 2006a; Grimm et al., 2009; Monici et al., 2011; Dittrich et al., 2018). While clinostat culture-induced angiogenesis is also recognized as an important factor of cardiovascular deconditioning faced by astronauts, it is inconsistent with the delayed wound healing during spaceflight in which neovascularization and ECM remodeling are impaired (Kirchen et al., 1995; Monici et al., 2011). On the other hand, the angiogenesis of ECs tends to be depressed in space experiments (Versari et al., 2013; Chakraborty et al., 2018). Thioredoxin-interacting protein is 33-fold up-regulated in ECs cultured in space, which can inhibit EC migration and impair angiogenesis (Versari et al., 2013). The gene expressions of eNOS and NO production are both suppressed in ECs exposed to space microgravity (Chakraborty et al., 2018). Both the type of ECs and the duration of the microgravity were comparable between the clinostat and spaceflight studies. These data indicate that the enhanced angiogenesis of ECs observed in clinostat culture may stem from other factors in the ground-based experiments except for distributing the gravity vector. As an example, shear stress could be one of the potential factors. While shear stress generated by clinostat can be minimized by placing the samples close to the center of rotation in RPM or optimizing initial speed of RWV, the influence of shear stress cannot be neglected especially when the rotational velocity is increased (Crabbé et al., 2008; Wuest et al., 2017) and the NO production is accumulated with culture duration. The production of NO by ECs cultured in RWV is dependent on the rotation rate, *that is*, 73%, 262%, and 500% increase is observed at 8, 15, and 20 rpm, respectively (Sanford et al., 2002). Taking into account that shear stress could induce NO production in ECs (Wuest et al., 2017), the enhanced NO release in the RWV experiment may originate from shear stress directly, implying the importance of mechanical parameters in clinostat studies. In fact, shear stress is a critical regulating factor in mass transfer for cell growth in space (Sun et al., 2008) or on ground (Cui et al., 2011) and highly associated with the relevant functions of the cells cultured. In current work, the shear stress only existed when the culture medium was refreshed, not persisted as the clinostat culture, and therefore the influence of shear stress should be less than that in the clinostat experiments.

In this work, space microgravity could suppress the release of a few pro-angiogenesis cytokines in ECs such as Endoglin, IGFBP-2, PDGF-AA, and Pentraxin-3, supporting the impaired angiogenesis as a previous work carried out in space mission (Versari et al., 2013). Decreased collagen I and fibronectin were also observed, consistent with the reduced ECM deposition in the hindlimb unloading experiments (Martinez et al., 2007). Impaired mechanotransduction through integrin-mediated signaling pathway led to decreased p-FAK and PI3K, which might result in the depressed expression of eNOS and be in agreement with previous spaceflight study (Chakraborty et al., 2018). The Cav-1 was also reduced by space microgravity, possibly counteracting with the decreased eNOS and causing the unchanged NO production.

This work provided several new findings about the effects of space microgravity on ECs. First, clinostat culture increased the expression of CD44 in thyroid cancer cells (Grosse et al., 2012). Here, CD44 on the surface of ECs was also up-regulated in space, which might lead to stronger signal of CD44-hyaluronan binding and increased vimentin through RhoA/ROCK (Bourguignon et al., 2006; Murray et al., 2014; Hyder et al., 2015). Second, vimentin is one of the intermediate filaments contributing to maintain the structural and mechanical stability of cells (Zhang et al., 2017; Zhou L.W. et al., 2018) and the increased vimentin expression is observed in papillary thyroid carcinoma cells and chondrocytes using clinostats (Infanger et al., 2006b; Aleshcheva et al., 2013). Here, vimentin is accumulated in ECs especially in the perinuclear region after long-term spaceflight (**Figure 6A** *3rd row*), which might compensate for the loss of F-actin and microtubules. Third, another enlightening finding is the enhanced mRNAs encoding actin and tubulin in exosomes under space microgravity. This could serve as a line of reasoning for the disorganization of F-actin and microtubules in ECs during spaceflight, revealing the potential of ECs to induce cytoskeletal remodeling in neighboring cells.

This study was restricted by a small sample size as previous space studies do (Chakraborty et al., 2014). We were only able to collect two sets of space samples (including the fixed cells at day 3 and day 10 and the corresponding supernatants) on board the SJ-10 satellite because of the resource limitations of the physical space and electrical power assigned to this project as well as the complexity of SCCS. To this end, we tried our bests to ensure robust statistical analyses, *that is*, the ground control experiments using same hardware were repeated four times independently and technical repetitions were performed for space and ground samples in each assay to reduce potential system errors and cellular heterogeneity. Nevertheless, the validation of the conclusions drawn from this study was required by future spaceflight experiments with larger sample sizes.

Hypergravity and vibrations derived from launch or landing phase and space radiation might interfere with the effects of microgravity in this study. On one hand, ECs exposed to hypergravity present the increased NO production, the enhanced cell migration and the altered cytoskeletal organization (Versari et al., 2007; Koyama et al., 2009). The downregulation of ACTB and ITBG1 genes are also observed in EA.hy926 cells under vibration (Wehland et al., 2013). These studies indicate that the effects of hypergravity and vibration may not be negligible. Nevertheless, these effects are hard to be isolated separately from the current space mission techniques, as the other related works do (Kapitonova et al., 2012). Moreover, these factors work in short-term duration (in minutes or hours) compared with long-term cell culture on orbit (in days), which could not be dominant environmental factors. On the other hand, space radiation is another potential interfering factor. For example, the synergistic expressions of pro-inflammatory cytokines, adhesive molecules and cytoskeletal proteins have been reported in ECs exposure to low- or high-dose radiation (Blirando et al., 2012; Choi et al., 2016; Soltani et al., 2016; Mao et al., 2018; Schröder et al., 2018). In the current work, the protection of the Earth’s magnetosphere and the satellite’s shell could resist most of the radiation since the SJ-10 satellite is running in a low-Earth orbit and exposed to space radiation at a low-dose rate of 0.064–0.075 mGy/d (Boerma et al., 2015; Liu et al., 2015; Delp et al., 2016; Zhou D.Z. et al., 2018), implying that the influence of radiation is less important in this study as other similar space mission assume (Versari et al., 2013; Chakraborty et al., 2014). Additional studies with appropriate in-flight 1 × *g* centrifuge control is required to isolate the effects of microgravity from other adverse factors of outer space flight in the future missions.

Due to the technical limitation in a single space mission of SJ-10 satellite, it is not able to elaborate the underlying mechanisms or mechanotransduction pathways in the effects of space microgravity on ECs. Nevertheless, our findings further the understandings of the difference between real microgravity and ground-based microgravity effect simulation on ECs, and provide helpful information for future spaceflight experimental design in EC-related functions.

## Author Contributions

NL and CW performed the experiments, analyzed the data, and wrote the manuscript. SS designed and prepared SCCS hardware. SS and CW implemented the preparations of launching and recovery in SJ-10 satellite mission. CZ, DL, and QC collected original space data and performed data processing. ML designed the study, supervised the experiments and analyses, and wrote the manuscript. All authors read and approved the final manuscript.

## Conflict of Interest Statement

The authors declare that the research was conducted in the absence of any commercial or financial relationships that could be construed as a potential conflict of interest.

## Footnotes

1 http://www.ncbi.nlm.nih.gov/sra/SRP149559
